# The Immune Heterogeneity Between Pulmonary Adenocarcinoma and Squamous Cell Carcinoma: A Comprehensive Analysis Based on lncRNA Model

**DOI:** 10.3389/fimmu.2021.547333

**Published:** 2021-07-29

**Authors:** Tao Yan, Guoyuan Ma, Kai Wang, Weidong Liu, Weiqing Zhong, Jiajun Du

**Affiliations:** ^1^Institute of Oncology, Shandong Provincial Hospital, Cheeloo College of Medicine, Shandong University, Jinan, China; ^2^Department of Thoracic Surgery, Shandong Provincial Hospital, Cheeloo College of Medicine, Shandong University, Jinan, China; ^3^Department of Interventional Radiology, Shandong Medical Imaging Research Institute Affiliated to Shandong University, Jinan, China; ^4^Department of Radiology, The Fourth People’s Hospital of Jinan, Jinan, China

**Keywords:** non-small cell lung cancer, squamous cell carcinoma, adenocarcinoma, immune, long non-coding RNAs

## Abstract

Adenocarcinoma (AD) and squamous cell carcinoma (SCC) are both classified as major forms of non-small cell lung cancer, but differences in clinical prognoses and molecular mechanisms are remarkable. Recent studies have supported the importance of understanding immune status in that it influences clinical outcomes of cancer, and immunotherapies based on the theory of “immune editing” have had notable clinical success. Our study aimed to identify specific long non-coding (lnc) RNAs that control key immune-related genes and to use them to construct risk models for AD and SCC. Risk scores were used to separate patients into high- and low-risk groups, and we validated the prognostic significance of both risk scores with our own cohorts. A Gene Set Enrichment Analysis suggested that the immune responses of patients in the AD high-risk group and the SCC low-risk group tended to be weakened. Evaluation of immune infiltration revealed that the degree of infiltration of dendritic cells is of particular importance in AD. In addition, prediction of responses to immune checkpoint inhibitor (ICI) treatments, based on the T Cell Immune Dysfunction and Exclusion and immunophenoscore models, indicated that deterioration of the immune microenvironment is due mainly to T cell exclusion in AD patients and T cell dysfunction in SCC patients and that high-risk patients with SCC might benefit from ICI treatment. The prediction of downstream targets *via* The Cancer Proteome Atlas and RNA-seq analyses of a transfected lung cancer cell line indicated that the lncRNA LINC00996 is a potential therapeutic target in AD.

## Introduction

Lung cancer ranks first in mortality among malignant tumors (1). More than 80% of lung cancer patients suffer from non-small cell lung cancer (NSCLC), which is classified into three pathological types: adenocarcinoma (AD), squamous cell carcinoma (SCC), and large cell carcinoma (2, 3). As the major pathological type of NSCLC, AD accounts for approximately 50% of NSCLC cases, while the share of SCC is approximately 40% (4, 5).

There are important differences between AD and SCC (1, 6). SCC is characterized by slower growth, a longer course, later metastasis, and higher 5-year survival rate after early surgery (7, 8). Accordingly, as compared with AD, the degree of malignancy of SCC is lower, and prognoses of patients tend to be better (1, 9–11). In recent years, with the rise of individualized treatment, more and more research has focused on the differences between AD and SCC (6, 12, 13). For instance, Lai-Goldman and colleagues have noted significant differences in tumor immunity among subtypes of AD and SCC (6). Thus, it can be seen that research on both AD and SCC is critical to the development of effective therapies for lung cancer.

The growing field of tumor immunology represents a possible source of therapies that can target characteristics of the diverse forms of NSCLC. From the proposal of “immune surveillance” in 1909 to the establishment of the theory of “immune editing” in 2014, the exploration of tumor immunology has been occurring for more than 100 years (14). Recent advances, though, have finally moved the field into clinical practice. Based on the state of human immunodeficiency during the tumor escape phase, many immune-checkpoint inhibitors (ICIs) have been introduced and have become mainstays of immunotherapy. These drugs include anti-programmed death-1 (PD-1) and anti-cytotoxic T-lymphocyte antigen 4 (CTLA-4) antibodies (15, 16). In particular, the combined application of ICIs and traditional radiochemotherapy has been shown to improve the survival rate of patients with lung cancer (15). However, relatively low tumor immunogenicity and other characteristics of the immunosuppressive tumor microenvironment that lead to insufficient T cell infiltration responses are a challenge in the field of ICI therapy (17–20).

To address these challenges, chemotherapy drugs based on “immunogenic cell death” (ICD), such as oxaliplatin and doxorubicin, have come into the fields of vision of researchers. ICD-based drugs induce antigen-presenting dendritic cells (DCs) to engulf dying tumor cells. Antigens presented by the DC then recruit cytotoxic T lymphocytes (CTLs) to kill the tumor cells (21, 22). ICD therapy has contributed to the enriching of the theory of immunotherapy, and, in the clinic, it has provided a mechanism to facilitate the killing of drug-resistant tumor cells, including those of NSCLC (23, 24).

The immune status of a tumor is likely to be a key factor influencing efficacy of immunotherapy, which in turn will affect the prognosis of the patient. According to the theory of immune editing, the mechanism of tumor survival during the escape phase involves several factors. One such factor is a weakened immune recognition, which can involve the loss of specific tumor antigens that are necessary to promote ICD. Another factor that may enhance survival at this point is the reinforcement of resistance or survival mechanisms, such as increased expression of signal transducer and activator of transcription 3 or the construction of an immunosuppressive tumor microenvironment. This latter case can involve the production of cytokines, such as vascular endothelial growth factor or transforming growth factor *β* or immunoregulatory molecules such as indoleamine 2,3-dioxygenase or PD-1/PD-L1, which are targets of ICIs (14).

Non-coding genes, which constitute 98.5% of the human genome, have been proven to play important roles in regulating many cellular processes (3), including immune functions. Long non-coding RNA (lncRNA) is a prominent product of non-coding genes and control gene expression at the levels of chromatin organization, transcription, and post-transcription (25). Various lncRNAs have been shown to be involved in various aspects of tumor immunology, including the immune microenvironment and immune cell infiltration (26–29). Thus, a lot of research has focused on roles of immune-related lncRNAs in multiple types of carcinomas, such as glioma, breast cancer, bladder cancer, and hepatocellular carcinoma (30–34). Sun et al. have also developed a model of immune-related lncRNAs in non-solid tumors (35). The achievements of these predecessors indicate the powerful potential and broad prospects of the immune-related lncRNA model.

Our research aims to identify significant immune-related RNAs in NSCLC from the Cancer Genome Atlas (TCGA) database to study differences of immune status between AD and SCC patients. Based on risk scores determined from a model based on expression of various lncRNAs, we compared populations of AD and SCC patients who would likely benefit from ICI treatment *via* their T cell immune dysfunction and exclusion (TIDE) and immunophenoscore (IPS). Furthermore, RNA-seq analysis and prediction of downstream protein targets were performed for notable lncRNAs.

## Materials and Methods

### Preparation and Downloading of Published Data

Files containing the clinical information and gene expression profiles of patients with AD and SCC were downloaded from TCGA (https://www.cancer.gov/about-nci/organization/ccg/research/structural-genomics/tcga/using-tcga/citing-tcga). Normal samples were excluded, leaving only cancer-related data for subsequent research. AD- and SCC-related proteomics data that corresponded to TCGA patient data were downloaded from the Cancer Proteome Atlas (TCPA) (https://tcpaportal.org/tcpa/credits.html).

The validation cohort consisted of 22 patients with SCC and 24 patients with AD, which all underwent surgery at Department of Thoracic Surgery, Shandong Provincial Hospital. The Biomedical Research Ethics Committee of Shandong Provincial Hospital approved this research (SWYX: NO.2020-256). It was performed based on the Declaration of Helsinki and Good Clinical Practice guidelines, as defined by the International Conference on Harmonization.

### Search for Immune-Related lncRNAs

The “Immune Response” gene set from Gene Set Enrichment Analysis (GSEA, https://www.gsea-msigdb.org/gsea/index.jsp) includes 235 genes. The function of those genes is to control immune processes to protect the organism from any biological threat. As a complement, there were 332 genes involved in the “Immune System Process” set, which additionally covers genes related to the development or functioning of the immune system. By correlation analysis to the genes from abovementioned sets, we selected the lncRNAs with a Person coefficient of more than 0.4 and a *p*-value less than 0.05 for model construction (32). Other lists of immune-related genes were download from Immport (https://www.immport.org/) (36) and The Cancer Immunome Atlas (TCIA).

### Survival Analysis and Construction of Risk Model

Only patients with OS more than one month were chosen for survival analysis, which was performed with the R package “Survival.” The clinical information of patients involved in the research is provided in **Supplementary Table S1**. After combining gene expression and clinical information, univariate and multivariate Cox analyses were performed to identify immune-related lncRNAs with prognostic significance, which made up the risk model. The risk score was calculated *via* this formula (30): $\text{Risk core}={\text{Σ}}_{i=1}^{n}\text{ coe}{\text{f}}_{i}*\text{lncRN}{\text{A}}_{\text{i}}\text{ expression}$

The boundary point of age was calculated by SPSS 24. Area Under Curve (AUC) and C-index calculations were performed with the R packages “survivalROC” and “survcomp”. The nomogram was built based on the R package “rms.” The principal component analysis (PCA) was performed with the R package “scatterplot3d”.

### Assessment of Immune Cell Infiltration Into Cancer Tissues

The fraction of immune cell was calculated by CIBERSORT algorithm (37). There were 22 types of immune cells involved in the evaluation: naïve B cells, memory B cells, plasma cells, CD8+ T cells, naïve CD4+ T cells, resting memory CD4+ T cells, activated memory CD4+ T cells, follicular helper T cells, regulatory T (Treg) cells, gamma delta T cells, resting natural killer (NK) cells, activated NK cells, monocytes, M0 macrophages, M1 macrophages, M2 macrophages, resting dendritic cells, activated dendritic cells, resting mast cells, activated mast cells, eosinophils, and neutrophils. The violin illustration was drawn with the R package “V”.

### Drawing of lncRNA-Gene and lncRNA–Protein Correlation Networks

A correlation analysis was performed as described above, except that when correlation of lncRNA between proteomes was involved, the standard was changed so that a coefficient greater than 0.3 and *p*-value less than 0.05 were considered relevant. The Sanquito diagram was constructed based on the R packages “ggplot2” and “ggalluvial.” The network was built with Cytoscape v3.7.1.

### Quantitative Real-Time PCR

Total RNA of carcinoid tissues was extracted with Trizol reagent (Lot A2A0209, ADurate Biotechnology, China) and evaluated *via* Nanodrop 2000 (Thermo Fisher Scientific, Waltham, MA, USA). A reverse transcription kit (A2A1386) was provided by ADurate Biotechnology (Human) Co. (China). The sequences of primers are in **Supplementary Table S2**. Real-time PCR was performed on a LightCycler 480 II (Roche, Basel, Switzerland) with the SYBR Green system (Lot A2A1436, ADurate Biotechnology).

### Cell Culture, Transfection, and RNA-Seq Analysis

NSCLC cell line A549 (Procell CL-0016) was provided by Procell Life Science & Technology Co. Cells were cultured in Roswell Park Memorial Institute 1640 media (RPMI 1640; Gibco, Waltham, MA, USA) supplemented with 10% fetal bovine serum (FBS Lot 1742862, Biological Industries, Beit HaEmek, Israel) and incubated at 37°C and 5% CO_2_. The transfection reagent was jetPRIME^®^ (Lot 22Y0302M15; Polyplus-transfection, Illkirch-Graffenstaden, France), and plasmids that direct the overexpression of LINC00996 (Ecoli_VB210422-1050dqh) and AP001189.3 (Ecoli_VB210422-1048dqh) were customized by VectorBuilder. After transfection, cellular RNA was extracted *via* Trizol, and samples were sent to Novogene Co., LTD (Beijing, China) for RNA-seq analysis. A detailed protocol is provided in **Supplementary Information**.

### Prediction of Response to ICI Treatment

IPS data of patients from the TCGA database was downloaded from TCIA, and this information was applied to quantify tumor immunogenicity. In this model, a higher score means that a patient was more likely to be sensitive to treatment with ICIs (38). T cell immune dysfunction and exclusion (TIDE) analysis was used to predict whether patients would benefit from ICIs based on CTL status, including T cell exclusion and T cell dysfunction (39). Here, a high score means a less favorable tumor immune environment (TIM). Preparation files were obtained from the University of California at Santa Cruz (UCSC) and normalized (40).

### Software and Statistical Analysis

All R packages were run under the R 3.6.1 (The R Foundation for Statistical Computing, Vienna, Austria) environment. Some statistical analyses were performed with SPSS 24 (SPSS Inc, Chicago, IL). Values of *p* < 0.05 were considered to be statistically significant.

## Results

### Immune-Related LncRNAs Found in NSCLC

**Figure 1:**
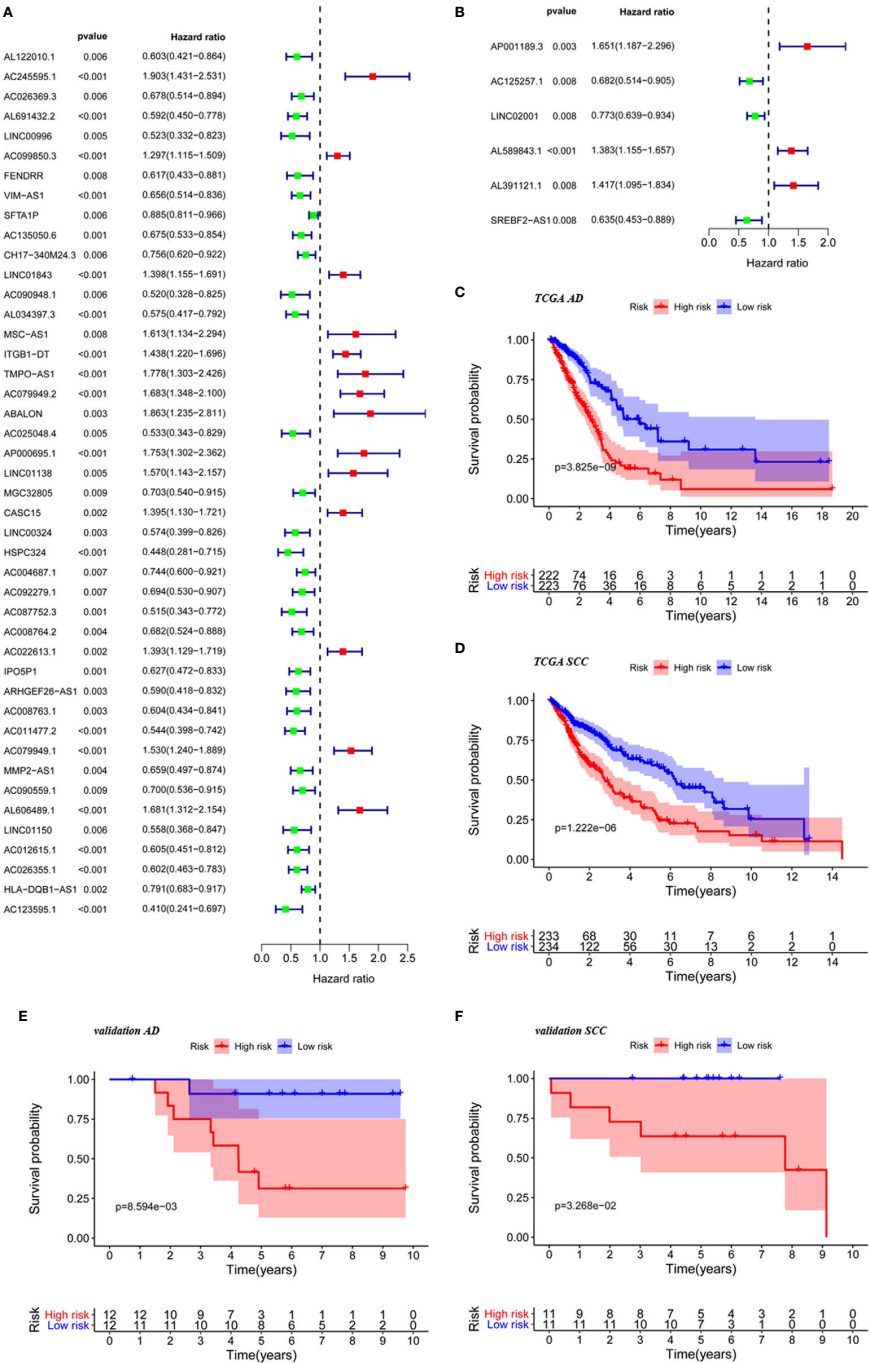
The construction of risk score based on significant lncRNAs. Immune-related lncRNAs selected by univariate Cox analysis for TCGA AD patients **(A)** and TCGA SCC patients **(B)**. KM curve for TCGA AD patients **(C)**, TCGA SCC patients **(D)**, AD patients from validation cohorts **(E)** and SCC patients from validation cohort **(F)** in low-risk group and high group based on the risk score.

In referring to the gene sets called “immune response” and “immune system progress” from GSEA, we searched for associated lncRNAs with Pearson correlation coefficients of at least 0.4 and values of *p* less than 0.05 in AD and SCC patients upon comparison of cancerous tissues to non-cancerous tissues. There were 1,124 eligible lncRNAs found in lung AD, and the number was reduced to 574 for SCC. We used a univariate Cox analysis and identified lncRNAs with significant prognostic value for the two main types of NSCLC (**Figures 1A, B**).

### Construction of Prognostic Model Based on Identified lncRNAs

A multivariate Cox analysis demonstrated that 12 LncRNAs for AD and 4 lncRNAs for SCC were fit to build the prognostic models (**Table 1**). A risk score was calculated according to the expression of these lncRNAs. The risk score for AD patients is defined according to the following equation, where relative lncRNA expression is noted with the relevant gene symbol: risk score (AD) = 0.3292 × AC245595.1 − 0.7741 × LINC00996 + 0.2592 × VIM − AS1 + 0.1354 × SFTA1P + 0.4720 × MSC − AS1 + 0.5041 * TMPO − AS1 + 0.4536 × ABALON + 0.36102 × AL606489.1 − 0.5293 × AC025048.4 + 0.5714 × LINC01138 − 0.2651 × IPO5P1 − 0.3861 × AC008763.1 − 0.3246 × AC026355.1 − 0.5747 × AC123595.1. The risk score for SCC patients is defined as 0.2936 × AP001189.3 + 0.2957 × AL589843.1 + 0.2409 × AL391121.1 − 0.3406 × SREBF2 − AS1.

**Table 1:** The significant LncRNAs screened *via* multiple Cox analysis for AD and SCC patients.

id	coef	HR	HR.95L	HR.95H	pvalue	cancer type
AC245595.1	0.329165921	1.389808435	1.032339779	1.871057887	0.030024749	AD
LINC00996	-0.774050359	0.461141492	0.274489195	0.77471711	0.003452225	
VIM-AS1	0.259247694	1.295954766	0.942574454	1.781820785	0.110510674	
SFTA1P	0.135422567	1.14502053	1.024648686	1.279533203	0.016865123	
MSC-AS1	0.472010646	1.603214451	1.098882381	2.339009724	0.014315352	
TMPO-AS1	0.504108967	1.65550975	1.078766249	2.540599072	0.021058957	
ABALON	0.453594618	1.573959813	0.965852294	2.564936179	0.068680403	
AC025048.4	-0.529348003	0.588988864	0.360466006	0.962387231	0.034600822	
LINC01138	0.571431248	1.770799691	1.226078075	2.557530072	0.002313998	
IPO5P1	-0.265163082	0.767080843	0.544994777	1.079667263	0.128400874	
AC008763.1	-0.386147513	0.679670258	0.452168184	1.021636806	0.063308276	
AL606489.1	0.361023263	1.434796838	1.081196869	1.904039889	0.012393827	
AC026355.1	-0.324603369	0.722813987	0.546493021	0.956023297	0.022894816	
AC123595.1	-0.574737554	0.562852568	0.330956606	0.957234294	0.033898149	
AP001189.3	0.293555222	1.34118724	0.935246211	1.923325848	0.110489673	SCC
AL589843.1	0.295670837	1.344027679	1.116320175	1.618183066	0.001797664	
AL391121.1	0.240903116	1.272397754	0.954266017	1.69658776	0.100783269	
SREBF2-AS1	-0.340606956	0.71133844	0.504721264	1.002538257	0.051716718	

Survival analysis using groups defined with these risk scores revealed that significant differences existed between high- and low-risk groups for AD (*p* < 0.001) and SCC (*p* < 0.001) as shown in **Figures 1C, D**. Further supporting the validity of these models, five-year survival rates for AD were 20.75% in the high-risk group and 50.10% in the low-risk group, and five-year survival rates for AD were 32.30% in the high-risk group and 60.70% in the low-risk group. Furthermore, the prognostic significance of the risk score also validated by analysis of our own AD and SCC cohorts (**Figures 1E, F**) (*p* < 0.05 in both cases).

**Figure 2:**
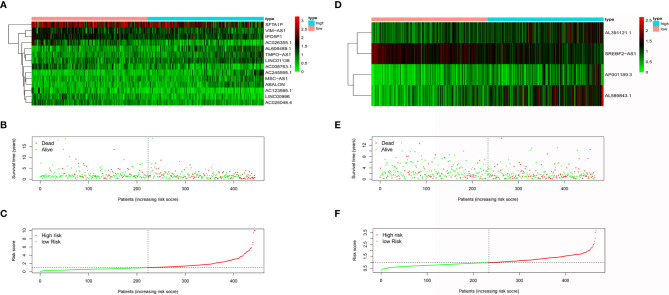
The effectiveness evaluation of risk score. Heatmap of model lncRNAs expression level for AD patients **(A)** and SCC patients **(D)** in low-risk group and high group. Survival condition of AD patients **(B)** and SCC patients **(E)** in low-risk group and high group. Risk score curve of AD patients **(C)** and SCC patients **(F)** in low-risk group and high group.

The populations were sorted from low to high according to the risk score and were divided into two groups (**Figures 2C, F**). The differences in expression of model lncRNAs are shown in **Figure 2A** for AD and **Figure 2D** for SCC. It is clear that some lncRNAs were enriched in the low-risk group. Enriched lncRNAs included IPO5P1, AC008763.1, AC026355.1, and AC123595.1 in AD and SREBF2-AS1 in SCC. Scatter plots identified that death toll increased with higher risk score, while the survival time was inversely related to risk score the (**Figures 2B, E**).

### Evaluation of Immune-Related lncRNA Models

**Figure 3:**
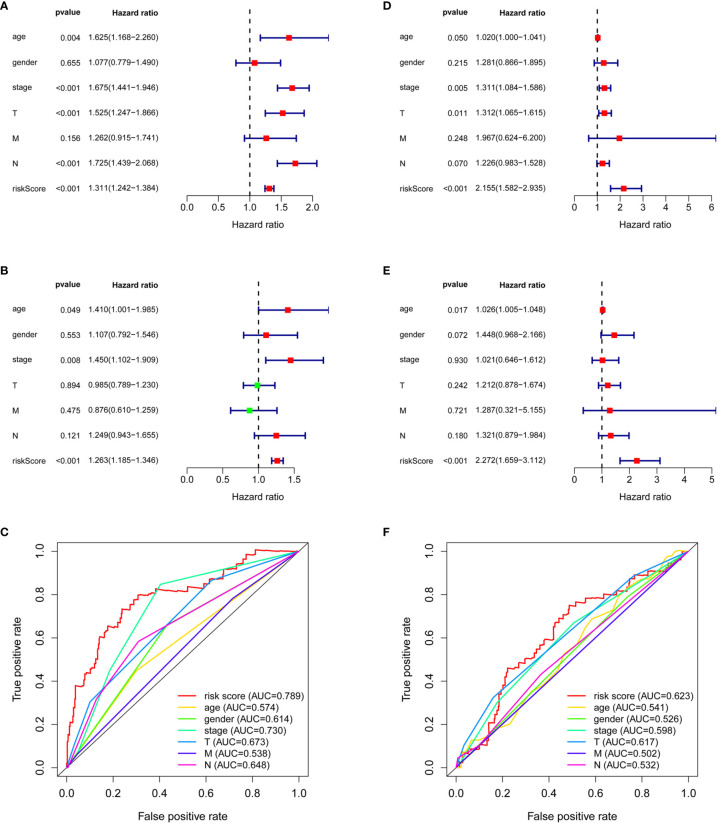
The comparison between risk score and clinical factors. Univariate Cox analysis on clinical factors and risk score for AD patients **(A)** and SCC patients **(D)**. Multivariate Cox analysis on clinical factors and risk score for AD patients **(B)** and SCC patients **(E)**. ROC curves of clinical factors and risk score for AD patients **(C)** and SCC patients **(F)**.

Although the Kaplan–Meier curve indicated significant differences in survival between the high-risk and low-risk groups (**Figures 1C, D**), we attempted to assess the predictive ability of the models in AD and SCC patients. A univariate Cox analysis showed that age (AD: *p* = 0.004, SCC: *p* = 0.050), stage (AD: *p* < 0.001, SCC: *p* = 0.005), tumor stage (AD: *p* < 0.001, p-value _SCC_=0.011), nodes (AD: *p* < 0.001) and risk score (AD: *p* < 0.001, SCC: *p* < 0.001) had prognostic significance to overall survival (**Figures 3A, D**). After multivariate analysis (**Figures 3B, E**), the included factors were limited to age (AD: *p* = 0.049, SCC: *p* = 0.017), stage (AD: *p* = 0.008), and risk score (AD: *p* < 0.001, SCC: *p* < 0.001).

It was surprising that the hazard ratio (HR) of the risk score in SCC patients was quite large: 2.155 (1.582–2.935, 95% CI) in the univariate analysis and 2.272 (1.659–3.112, 95% CI) in the multivariate analysis. The HR of the risk score in AD patients was relatively small, 1.311 (1.242–1.384, 95% CI) by univariate analysis and 1.263 (1.185–1.346, 95% CI) by multivariate analysis, even though there were additional independent prognostic factors, such as age and stage, for AD. These additional factors had strong HR values, 1.410 (1.001–1.985, 95% CI) and 1.450 (1.102–1.909, 95% CI), but the large confidence intervals weakened their impact (**Figures 3B, E**).

Importantly, the risk score possessed the highest AUC, 0.789 in AD patients and 0.623 in SCC patients, as compared with age (AUC_AD_ = 0.574, AUC_SCC_ = 0.541), gender (AUC_AD_ = 0.614, AUC_SCC_ = 0.526), stage (AUC_AD_ = 0.730, AUC_SCC_ = 0.598), tumor stage (AUC_AD_ = 0.673, AUC_SCC_ = 0.617), nodes (AUC_AD_ = 0.648, AUC_SCC_ = 0.532), and metastases (AUC_AD_ = 0.538, AUC_SCC_ = 0.502) (**Figures 3C, F**). The C-index of the risk score was 0.712 (95% CI: 0.663–0.760, *p* < 0.001) in AD patients and was 0.616 (95% CI: 0.571–0.661, *p* < 0.001) in SCC.

### Application of the Model in Clinical Prediction and Immune Status

**Figure 4:**
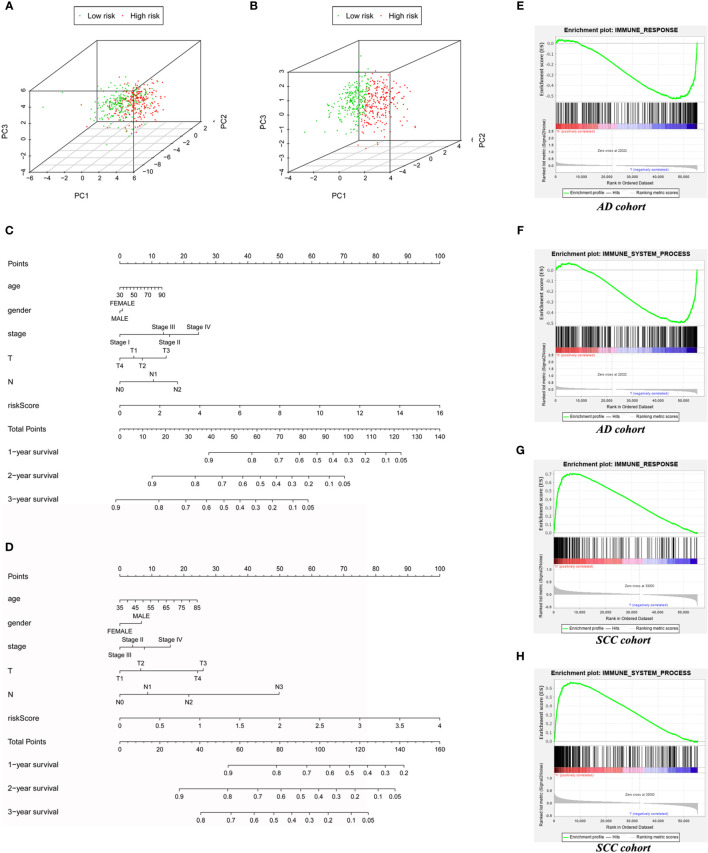
The expand application of risk score in prognostic prediction and immune status assessment. The PCA based on AD immune-related LncRNAs model **(A)** and SCC’s **(B)** to test the discrimination between low-risk and high-risk patients. **(C)** The Nomogram for AD cohort **(C)** and SCC cohort **(D)** to predict 1-year, 2-year and 3-year prognosis. **(E)** The GSEA based on “immune response” gene set for AD patients. **(F)** The GSEA based on “immune system process” gene set for AD patients. **(G)** The GSEA based on “immune response” gene set for SCC patients. **(H)** The GSEA based on “immune system process” gene set for SCC patients.

Compared with protein coding genes, immune-related genes and immune-related lncRNAs (**Supplementary Figure S1**), model lncRNAs held a stronger ability to distinguish high- and low-risk groups, which was demonstrated by PCA analysis of AD patients (**Figure 4A**) and SCC patients (**Figure 4B**). To expand the clinical applications of the model and predict the 1- to 3-year survival rate of lung cancer patients, we therefore combined age, stage, and other factors to build a nomogram on the basis of risk score (**Figures 4C, D**).

Interestingly, although immune-associated GSEA enrichment analyses of the “immune system process” set (AD: *p* = 0.029, SCC: *p* < 0.001) and “immune response” set (AD: *p* = 0.046, SCC: *p* < 0.001) demonstrated statistical significance in comparison to high- and low-risk groups of both AD and SCC patients, the actual results in these two carcinomas were in opposition. In the low-risk group of AD patients, the two sets of genes, immune response, and immune system, were enriched (**Figures 4E, F**). In contrast, these sets of genes were enriched in the high-risk group of SCC patients (**Figures 4G, H**). Thus, the role of the immune system in different tumors seems to vary, even if the primary organs are same.

**Figure 5:**
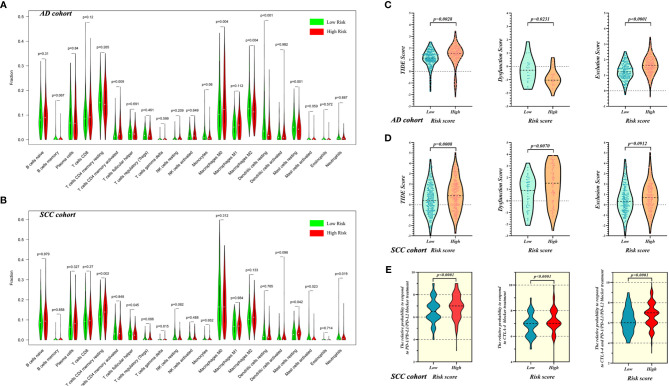
The evaluation of immune microenvironment in high- and low-risk patients with AD and SCC. The evaluation of immune cell infiltration for AD cohort **(A)** and SCC cohort **(B)**. The evaluation of tumor immune microenvironment based on TIDE for AD cohort **(C)** and SCC cohort **(D)**, and the grading system contain TIDE score, T cell Dysfunction Score and Exclusion Score. **(E)** The prediction of response to ICI treatment for SCC patients in high- and low-risk groups.

Analysis with the CIBERSORT program revealed that the distribution of resting dendritic cells (*p* < 0.001), activated memory CD4 T cells (*p* = 0.009), M0 macrophages (*p* = 0.004), M2 macrophages (*p* = 0.004), and resting mast cells (*p* = 0.001) had statistical significance in AD samples (**Figure 5A**). However, for SCC patients, only resting memory CD4 T cells (*p* = 0.002), follicular helper T cells (*p* = 0.045), resting mast cells (*p* = 0.042), activated mast cells (*p* = 0.023), and neutrophils (*p* = 0.019) were worthy of note (**Figure 5B**).

In addition to evaluation of infiltrated immune cells, we used TIDE to explore the differences of immune status between high- and low-risk groups of AD and SCC patients. For AD patients, **Figure 5C** shows that the TIM of the high-risk group was worse than the low-risk group (mean TIDE score of high-risk = 1.354, mean TIDE score of low-risk = 1.102; *p* = 0.0028) and that T cell exclusion was the main reason for the difference (*p* < 0.0001). Similarly, SCC patients in the high-risk group possessed worse TIM than did patients in the low-risk group (mean TIDE score of high-risk = 0.9588, mean TIDE score of low-risk = 0.5061; *p* = 0.0008), but here, T cell dysfunction was the main reason contributing to the difference (*p* = 0.0070, **Figure 5D**).

Furthermore, as shown in **Figure 5E**, the prediction of responses to ICI treatment showed that compared with low-risk SCC patients, high-risk patients with SCC would be more sensitive to PD-1/PD-L1/PD-L2 blocker (IPS of high-risk = 6.961 and IPS of low-risk = 6.556; *p* < 0.0001), CTLA-4 blocker (IPS of high-risk = 7.262 and IPS of low-risk = 6.850; *p* < 0.0001) and combination therapy of both (IPS of high-risk = 6.738 and IPS of low-risk = 6.197; *p* < 0.0001).

Cross table analysis indicated that more patients at stages II, III and IV were in the high-risk group in the AD cohort, while the patients in stage I were significantly more likely to be assigned to the low-risk group (*p* < 0.05, **Supplementary Table S3**). However, this potential clinical relevance was not seen in SCC patient data (**Supplementary Table S4**).

### Clinical Relevance of Model lncRNAs

The above results demonstrated the practicability and effectiveness of the prognostic model. Next, our work focused on the exploration of the individual lncRNAs constituting the model.

**Figure 6:**
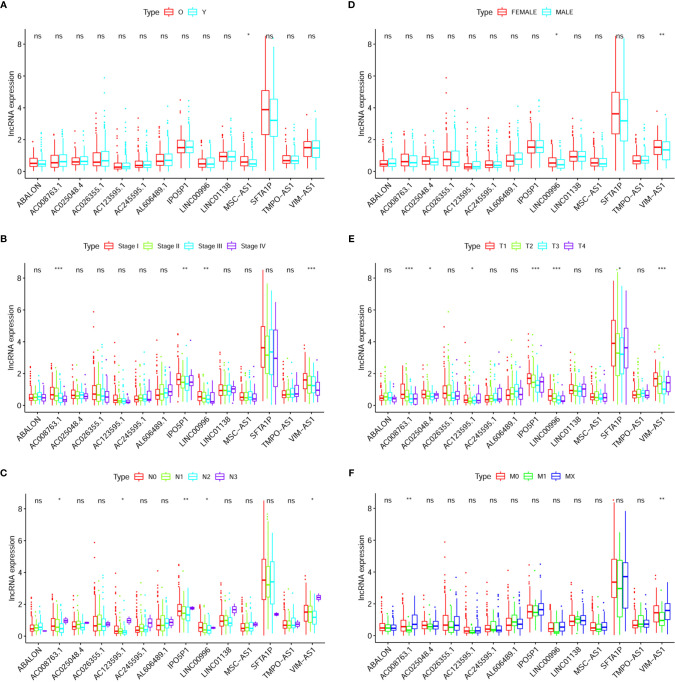
Clinical correlation analysis between expression of LncRNA and prognostic factors of AD patients: Age **(A)**, Gender **(B)**, Stage **(C)**, T **(D)**, N **(E)**, M **(F)**. The “*” means that p < 0.05. The “**” means that p < 0.01. The “***” means that p < 0.001; ns, no significance.

AD-related results are shown in **Figure 6**. **Figure 6A** exhibited that the expression of MSC-AS1 (*p* < 0.05) was lower in a group consisting of older patients than in the younger group. In females, expression levels of LINC00996 (*p* < 0.05) and VIM-AS1 (*p* < 0.01) were higher (**Figure 6B**). The data displayed in **Figure 6C** indicates that the expression levels of AC008763.1 (*p* < 0.001), IPO5P1 *(p <* 0.05), LINC00996 *(p <* 0.05), and VIM-AS1 (*p* < 0.001) were negatively related with rising stage, though it should be noted that due to the small number of samples at tumor stage 4, bias might have occurred. The expression of AC008763.1 (*p* < 0.001), AC025048.4 (*p* < 0.05), AC123595.1 (*p* < 0.05), IPO5P1 (*p* < 0.001), LINC00996 (*p* < 0.001), SFTA1P (*p* < 0.05) and VIM-AS1 (*p* < 0.001) tended to decrease as the tumor progressed, with the data from tumor stage 4 ignored (**Figure 6D**).

As the N stage changes (N3 samples were rare and not included), the difference in expression of these RNAs was statistically significant: AC008763.1 (*p* < 0.001), AC123595.1 (*p* < 0.05), IPO5P1 (*p* < 0.01), LINC00996 (*p* < 0.05), and VIM-AS1 (*p* < 0.05) (**Figure 6E**). The expression levels of AC008763.1 (*p* < 0.01) and VIM-AS1 (*p* < 0.01) were significantly lower in the M1 group than in the M0 group and MX group (**Figure 6F**).

In SCC patients, only the expression of AL589843.1 was positively correlated with cancer metastasis *(p <* 0.05; **Supplementary Figure S2E**). Other differences were not statistically significant.

### RNA-Seq Analysis Based on A549 Cells Overexpressing LINC00996

The correlation between model lncRNAs and immune-related gene is exhibited in **Supplementary Figure 3** for SCC patients and **Supplementary Figures S4**, **S5** for AD patients. According to the numbers of correlated genes, long intergenic non-protein coding RNA 996 (LINC00996) was selected for proteomics analysis in AD. As shown in **Supplementary Figure S5**, PD-L1, LCK, and SYK were potential targets of LINC00996 (cor > 0.3, *p* < 0.001).

**Figure 7:**
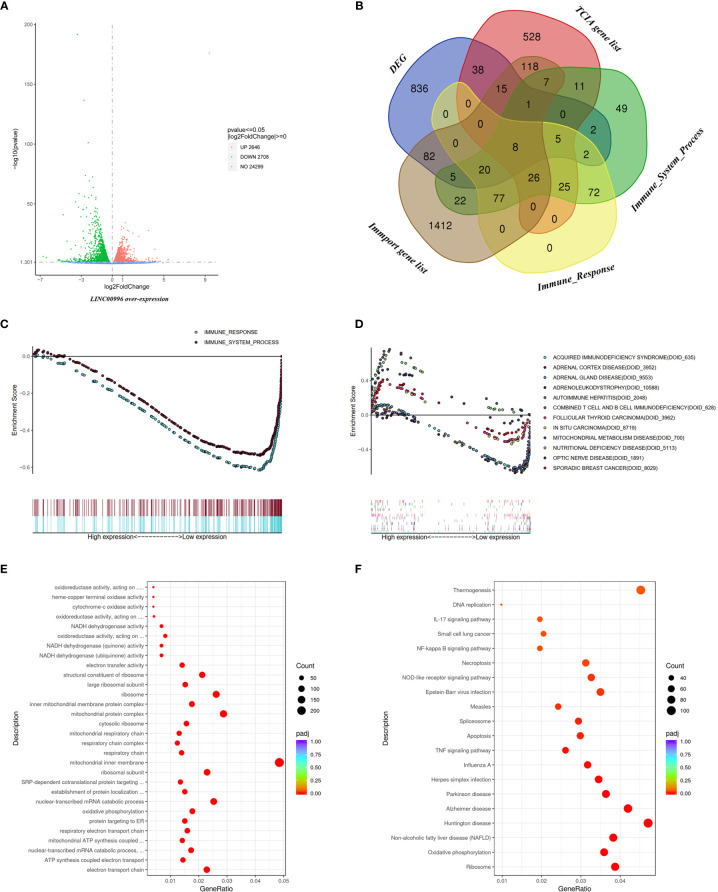
Functional analysis based on RNA-seq data. **(A)** The DEGs between A549 with over-expressed LINC00996 and control group exhibited *via* Volcano diagram. **(B)** The intersection between DEGs, TCIA gene list, Immune_System_Process from GSEA, Immune_Response from GSEA and Immport gene list exhibited *via* Venn diagram. Enrichment analyses including immune-related gene sets **(C)** and Disease Ontology **(D)** *via* GSEA analysis, Gene Ontology **(E)** and Kyoto Encyclopedia of Genes and Genomes term **(F)** based on the DEGs between A549 with over-expressed LINC00996 and control group.

Given that LINC00996 had shown potential as a target for AD treatment, we extracted total RNA from transfected A549 overexpressing LINC00996 (**Supplementary Figure S6**) and performed RNA-seq analysis with PC3.1-transfected A549 as a control. Extractions and analyses of both the control group and the experimental group were performed in triplicate. There were 1,014 differentially expressed genes (DEGs) found; 639 of these DEGs were downregulated (**Figure 7A**). We downloaded immune-related gene lists from TCIA, Immport, and GSEA, and a Venn diagram analysis indicated that 178 DEGs were immune-related (**Figure 7B**). Based on gene sets “immune response” and “immune system progress,” GSEA enrichment analysis demonstrated that the expression level of LINC00996 strongly influenced immune pathways in AD cells (*p* < 0.001 for both gene sets; **Figure 7C**).

In order to explore the value of LINC00996 value beyond cancer and immunology, Disease Ontology enrichment was performed, and the result showed that in addition to immune diseases (DOID_628 and DOID_635), genes related to adrenal diseases (DOID_3952, DOID_9553 and DOID_10558) were also enriched (**Figure 7D**). Meanwhile, Gene Ontology analysis indicated that the DEGs were focused on pathways involving energy metabolism, such as ATP synthesis and NADH dehydrogenation (**Figure 7E**). The result of Kyoto Encyclopedia of Genes and Genomes pathway analysis showed that thermogenesis and neurological diseases such as Parkinson or Alzheimer diseases might also be connected to LINC00996 (**Figure 7F**). Full enrichment results are provided in **Supplementary Information**.

## Discussion

The incidence of AD and SCC is very high in patients with lung cancer (1). Accordingly, research into new clinical treatments has been receiving much attention. Many articles have been published regarding various aspects of relationships of lncRNA models to NSCLC. For example, Sun and co-workers screened lncRNA expression from large NSCLC cohorts, including AD, SCC, and large cell carcinoma patients (41). This research was a significant effort, and the resulting lncRNA model has wide applicability. In our initial vision, although AD and SCC both belong to NSCLC, there are significant differences in pathogenesis and clinical traits that have led to discrepancies in the effectiveness of specific targeted drugs (6, 8). Therefore, we separated AD and SCC samples during this research and explored the differences between those two subtypes.

In general, patient cohorts tend to come from TCGA database, and researchers frequently supplement data sets with GEO data (42, 43). However, the gene expression matrix from TCGA generally possesses more lncRNAs than do GEO datasets because of many unnamed lncRNAs, such AP001189.3 present in TCGA, and other lncRNAs identified based on tentative research are only included in TCGA files (31, 44, 45). In order to include as many lncRNAs in our model as possible, we screened lncRNAs from TCGA and used our own cohorts to validate results *via* real-time PCR. Recent articles related to lncRNA models put emphasis on the selection of the population, such as patients in early stage (42) or with a certain clinical trait such as relapse (46), and such constructed models have possessed excellent clinical value. Given the importance of immune status and application of immune therapy, we emphasized the status of the immune system to construct our lncRNA-related risk model.

Multivariate Cox analysis showed that the risk score of our model was an excellent independent prognostic factor for NSCLC patients. The prediction model has a satisfactory ability to distinguish high- and low-risk groups, which was shown in Kaplan–Meier plots. AUC and C-index value analyses also supported the reliability of the model. The above results strongly suggest that the prognostic model composed of immune-related lncRNAs has high clinical value.

In addition to clinical applications, we are also interested in understanding the immune status in lung cancer because the effectiveness of immunotherapy is based on the identification of the immune status of patients. According to the theory of immune editing, a patient’s own immune system can no longer effectively identify and kill tumors during the escape phase (14). Both ICI and ICD are expected to improve the immune response so that the immune system can perform its functions effectively. Therefore, the evaluation of immune status was necessary, and we performed the assessment *via* three methods: GSEA analysis, calculation infiltrated fraction of immune cells, and TIDE. Furthermore, other research studies concerning immune-related lncRNA models that have studied immunotherapy applications have stopped at the expression level of immune biomarkers, such as PDL1 or CTLA-4 (32, 34, 43). On the other hand, we used a system scoring method combined with TIDE to predict response of patients to ICIs.

The GSEA analysis of “immune response” and “immune system” suggested that there were statistically significant differences (*p* < 0.05) regarding the enrichment results of immune-related gene sets between high- and low-risk groups for both AD and SCC. Although the outcome proved that the model successfully combined immune features with clinical outcomes, we were particularly impressed with the diversity between AD and SCC. For example, in the AD cohort, immune-related gene sets were both enriched in the low-risk group, but the result was reversed in the SCC cohort. We hypothesize that the enrichment of genes sets represents an active immune response. However, our results show that the situation in AD and SCC should be analyzed and treated separately.

For AD patients, the weakness of the immune response in the high-risk group corresponds to the “escape phase” aspect of the theory of “immune editing.” Immunosuppressive tumor microenvironment or absence of antigen recognition may lead to this cold immune response. Considering the results of analyses of the degree of immune cell infiltration, we demonstrated that the distribution of DC cells (including professional antigen-presenting cells, the important part of the progress of immunogenic cell death) was statistically significantly different and that the fraction of immunosuppressive cells, such as Treg, changed only a little between high- and low-risk groups.

Recognition of tumor antigens is a key factor that induces the immune response. With the development of tumors and the elimination of the immune system, identifiable antigens are reduced and antigen presentation ability becomes weaker. Ultimately, unrecognized antigens remain, and endogenous presentation pathways fail because of the deletion of major histocompatibility complex I. Additionally, compared with the low-risk group, more patients in the high-risk group were at stage III or IV. This indicates that AD patients are more prone to immune escape in the late stage of tumor progression. In response to this phenomenon, ICD-based chemotherapeutics may have better therapeutic effects in the high-risk group because they adjust the presentation of antigen to emphasize exogenous presentation and they thus reactivate the immune response of the body. Correspondingly, the prediction of response to ICI treatment based on IPS for AD patients indicated a negative result between high- and low-risk groups (**Supplementary Figure S7**). The high number of infiltrating DC cells, which play a key role in ICD progression, in the low-risk group samples also supports this view.

The opposite results in SCC patients suggest that the variability of the SCC-associated antigens may be weak, or that the immune response is not strongly suppressed in the tumor microenvironment. The progression of carcinoma would remain under the control of the immune system. This may be one of the reasons why the prognosis of SCC patients is better than that of AD patients (8).

The TIDE evaluation for AD and SCC was also contradictory. Though both AD and SCC patients in high-risk group possessed higher TIDE scores and worse TIM than patients in low-risk group, the mechanism of deterioration was different. When CTL was relatively insufficient, the TIDE score was equal to the T cell exclusion score, because the exclusion effect of the TIM was the main factor causing the decrease in the number of CTL cells and immunosuppression (47). For the AD cohort, the T cell exclusion score of high-risk patients was significantly higher than that of low-risk patients (**Figure 5C**). Though the T cell dysfunction score was opposite, the overall TIDE score was significantly higher in the high-risk group. Therefore, T cell exclusion was the main factor for the ITM of AD patients, and this exclusion corresponded to the lower numbers of DC cells and lower immune response mentioned above.

As for SCC patients, the T cell dysfunction score was the main factor for ITM (**Figure 5D**), which reflected the fact that the number of CTLs in the TIM was sufficient but subject to functional obstruction. The aim of ICI treatment is to lift the restriction of tumor cells on CTL, and the prediction of response indicated that the high-risk patients were more likely to benefit from ICI treatments, including PD-1/PD-L1/PD-L2 blocker, CTLA-4 blocker and combination therapy, compared with low-risk patients (**Figure 5E**).

While we conducted clinical research, we also focused on lncRNAs that can be applied to basic studies. Among the lncRNAs selected for the AD model, LINC00996 is undoubtedly the most notable. Its HR was very low at only 0.5, which means significant protection for patients. LINC00996 was closely correlated with clinical indicators, such as stage (*p* < 0.01), T (*p* < 0.01), and N (*p* < 0.05). The results suggested that the expression of LINC00996 decreased with the malignancy of the tumor. Meanwhile, changes of the independent prognostic factor age did not influence the expression of LINC00996, which suggested that the protective significance of this gene might be suitable for patients of all ages. Compared with men, women have higher levels of LINC00996 expression (*p* < 0.05). The most surprising finding, however, is that it is co-expressed with 75 immune-related genes (cor value > 0.4), far exceeding other lncRNAs. The data from TCPA showed that PD-L1, LCK, and SYK were particularly strongly correlated with LINC00996 (cor > 0.3, *p* < 0.001). As an important target of ICIs, PD-L1 was discussed above. SYK is a non-receptor tyrosine kinase that regulates classical immunoreceptors, such as the B-cell receptor. LCK is another non-receptor tyrosine kinase that plays an essential role in the selection and maturation of developing T cells in the thymus and in the function of mature T cells (information of SYK and LCK from Uniprot, https://www.uniprot.org/uniprot/) (48).

The RNA-seq analysis based on A549 with overexpressed LINC00996 indicated that DEGs were closely related to the immune pathway and were enriched in immune gene sets (**Figures 7B, C**). Thus, the genomic and proteomic analyses strongly suggest that LINC00996 is a potential target of tumor immunology. Furthermore, function enrichment result of Disease Ontology, Gene Ontology and Kyoto Encyclopedia of Genes and Genomes analyses indicated that the potential value of LINC00996 goes beyond lung cancer and immunology. In SCC samples, the Sankey diagram proved that the lncRNA with the most co-expressed genes was AP001189.3 (**Supplementary Figure S5**). However, we did not find potential targets in the TCPA database for AP001189.3 (data not shown). The raw data of RNA-analysis for AP001189.3 is provided in **Supplementary Information**.

In this research, we attempted to construct an immune-related clinical model for NSCLC patients with notable lncRNAs. In addition, for significant RNAs, we utilized the protein database TCPA to analyze potential targets. In SCC, the high-risk group of patients as defined by our model had a higher degree of immune response, while the low-risk group had a higher immune response in the case of AD. This divergence provides a starting point for research into the immunology and immunotherapy of different subtypes of NSCLC.

The limitations of this research include the exclusion of GEO data, which were not used because the GEO datasets are missing necessary lncRNAs. Secondly, it should be noted that the number of proteins detected in the TCPA database was limited. Finally, conclusions drawn from results of bioinformatics analyses have not yet been verified by *in vitro* and *in vivo* biological experiments.

## Data Availability Statement

The datasets presented in this study can be found in online repositories. The names of the repository/repositories and accession number(s) can be found below: GEO, GSE179934.

## Ethics Statement

All human data were published and download in the TCGA and TCPA database. The validation cohort consisted of 22 patients with SCC and 24 patients with AD, which all underwent surgery at Department of Thoracic Surgery, Shandong Provincial Hospital. The Biomedical Research Ethics Committee of Shandong Provincial Hospital approved this research (SWYX: NO.2020-256). The patients/participants provided their written informed consent to participate in this study.

## Author Contributions

TY and GM conceived and designed the research, collected and analyzed the data, contributed reagents/materials/analysis tools, prepared figures and/or tables, and authored or reviewed drafts of the paper. KW contributed reagents/materials/analysis tools, prepared figures and/or tables, and authored or reviewed drafts of the paper. WL analyzed the data, contributed reagents/materials/analysis tools, and prepared figures and/or tables. WZ contributed reagents/materials/analysis tools. JD conceived and designed the research, and provided the guidance for analysis. All authors contributed to the article and approved the submitted version.

## Funding

The work was supported by National Natural Science Foundation 81672288 (to JD), Focus on Research and Development Plan in Shandong Province 2018GSF118028 (to JD) and Natural Science Foundation of Shandong Province ZR2019PH002 (to KW).

## Conflict of Interest

The authors declare that the research was conducted in the absence of any commercial or financial relationships that could be construed as a potential conflict of interest.

## Publisher’s Note

All claims expressed in this article are solely those of the authors and do not necessarily represent those of their affiliated organizations, or those of the publisher, the editors and the reviewers. Any product that may be evaluated in this article, or claim that may be made by its manufacturer, is not guaranteed or endorsed by the publisher.

## Supplementary Material

Supplementary Figure 1 **(A)** The PCA based on protein coding genes of AD patients. **(B)** The PCA based on immune-related genes of AD patients. **(C)** The PCA based on immune-related lncRNAs of AD patients. **(D)** The PCA based on protein coding genes of SCC patients. **(E)** The PCA based on immune-related genes of SCC patients. **(F)** The PCA based on immune-related lncRNAs of SCC patients.

Supplementary Figure 2 Clinical correlation analysis between expression of LncRNA and prognostic factors of SCC patients: Age **(A)**, Gender **(B)**, Stage **(C)**, T **(D)**, N **(E)**, M **(F)**. The “*” means that p<0.05. The “**” means that p<0.01. The “***” means that p<0.001.

Supplementary Figure 3 Sankey diagram to show the correlation ship between model lncRNAs and immune related genes in SCC patients.

Supplementary Figure 4 Sankey diagram to show the correlation ship between immune related genes and model lncRNAs except Linc00996 in AD patients.

Supplementary Figure 5 Potential immune-related targets from TCPA (green round rectangle) and TCGA (orange ellipse) database of Linc0996 (blue ellipse).

Supplementary Figure 6 PCR validated the achievement of LINC00996 and AP001189.3 transfection.

Supplementary Figure 7 The insignificant prediction result of ICI treatment between AD patients in high- and low-risk group.
