# Supplementary figures and images for: The Immune Heterogeneity Between Pulmonary Adenocarcinoma and Squamous Cell Carcinoma: A Comprehensive Analysis Based on lncRNA Model
Source: Front Immunol. 2021 Jul 29;12:547333. doi: 10.3389/fimmu.2021.547333 (PMC8358782; doi:10.3389/fimmu.2021.547333)

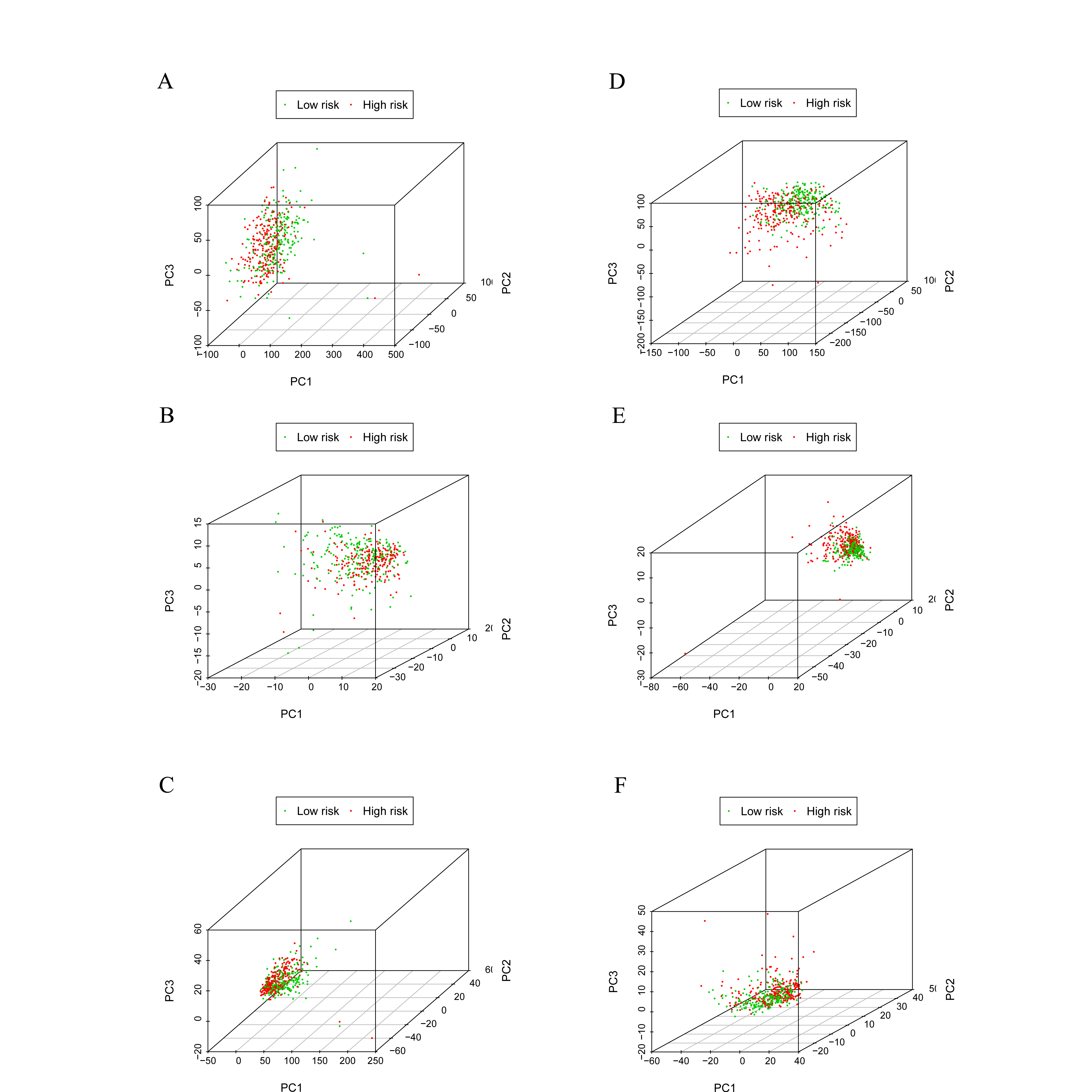

Supplement: Supplementary Figure 1 — (A) The PCA based on protein coding genes of AD patients. (B) The PCA based on immune-related genes of AD patients. (C) The PCA based on immune-related lncRNAs of AD patients. (D) The PCA based on protein coding genes of SCC patients. (E) The PCA based on immune-related genes of SCC patients. (F) The PCA based on immune-related lncRNAs of SCC patients. [file Image_1.jpeg]

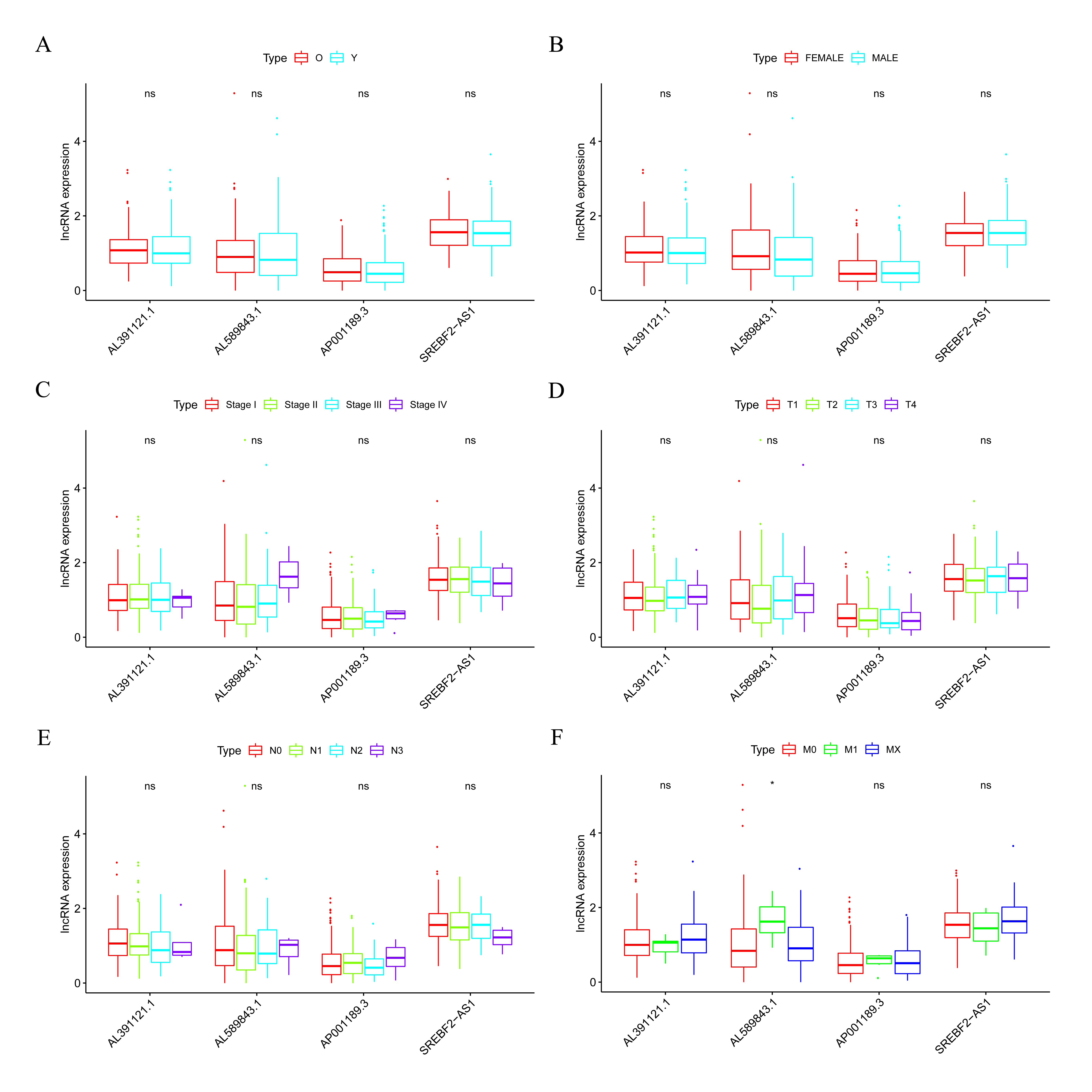

Supplement: Supplementary Figure 2 — Clinical correlation analysis between expression of LncRNA and prognostic factors of SCC patients: Age (A), Gender (B), Stage (C), T (D), N (E), M (F). The “*” means that p<0.05. The “**” means that p<0.01. The “***” means that p<0.001. [file Image_2.jpeg]

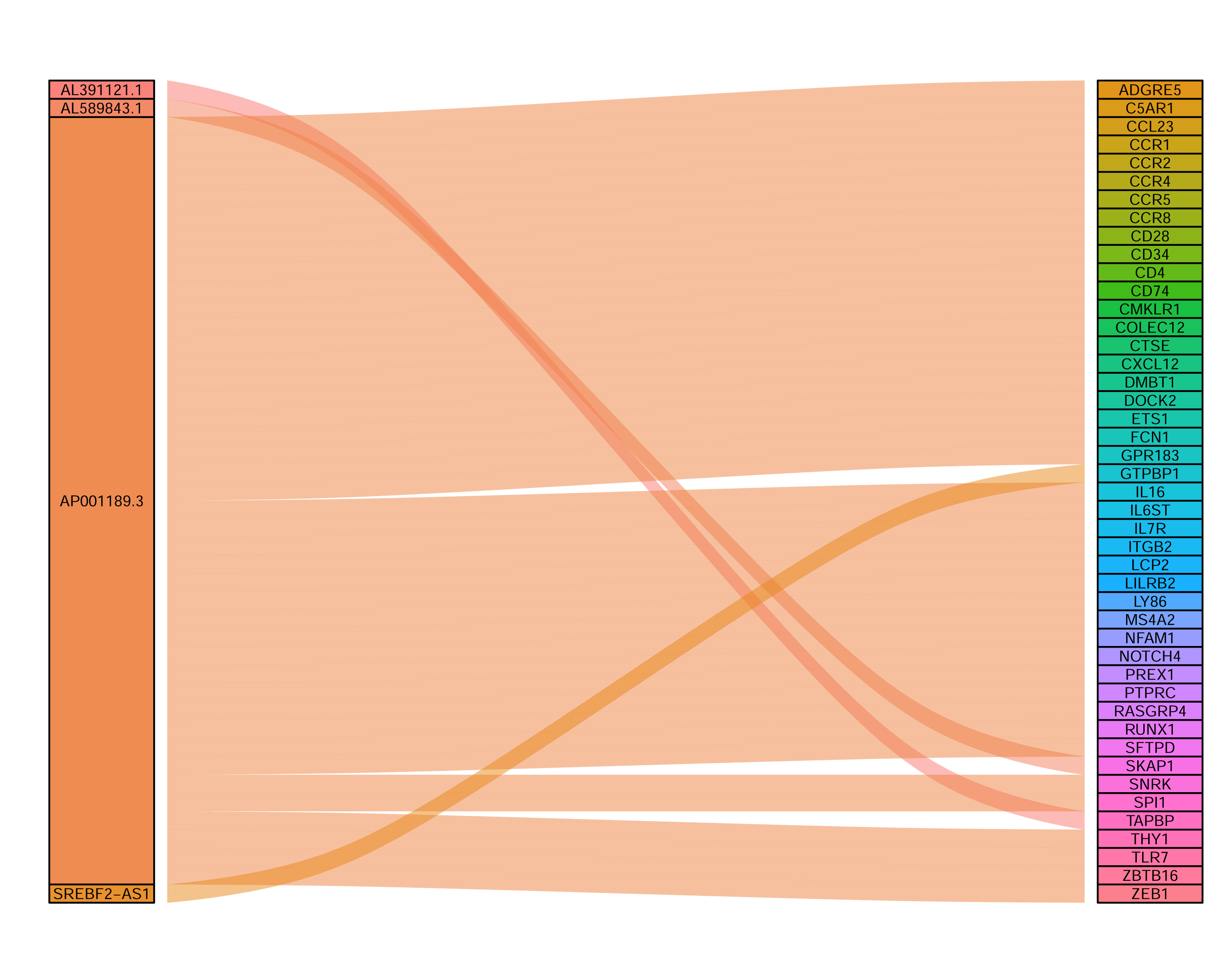

Supplement: Supplementary Figure 3 — Sankey diagram to show the correlation ship between model lncRNAs and immune related genes in SCC patients. [file Image_3.jpeg]

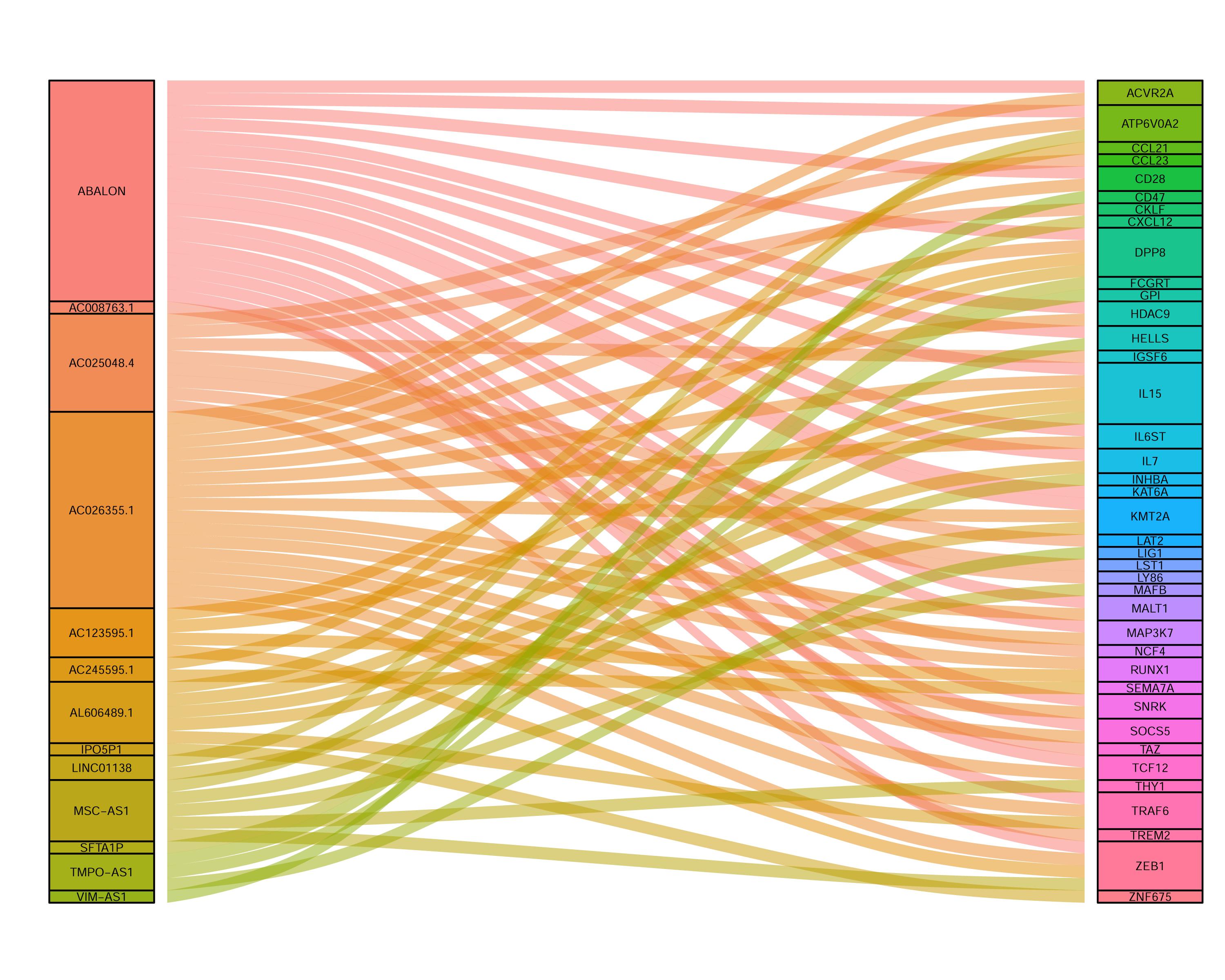

Supplement: Supplementary Figure 4 — Sankey diagram to show the correlation ship between immune related genes and model lncRNAs except Linc00996 in AD patients. [file Image_4.jpeg]

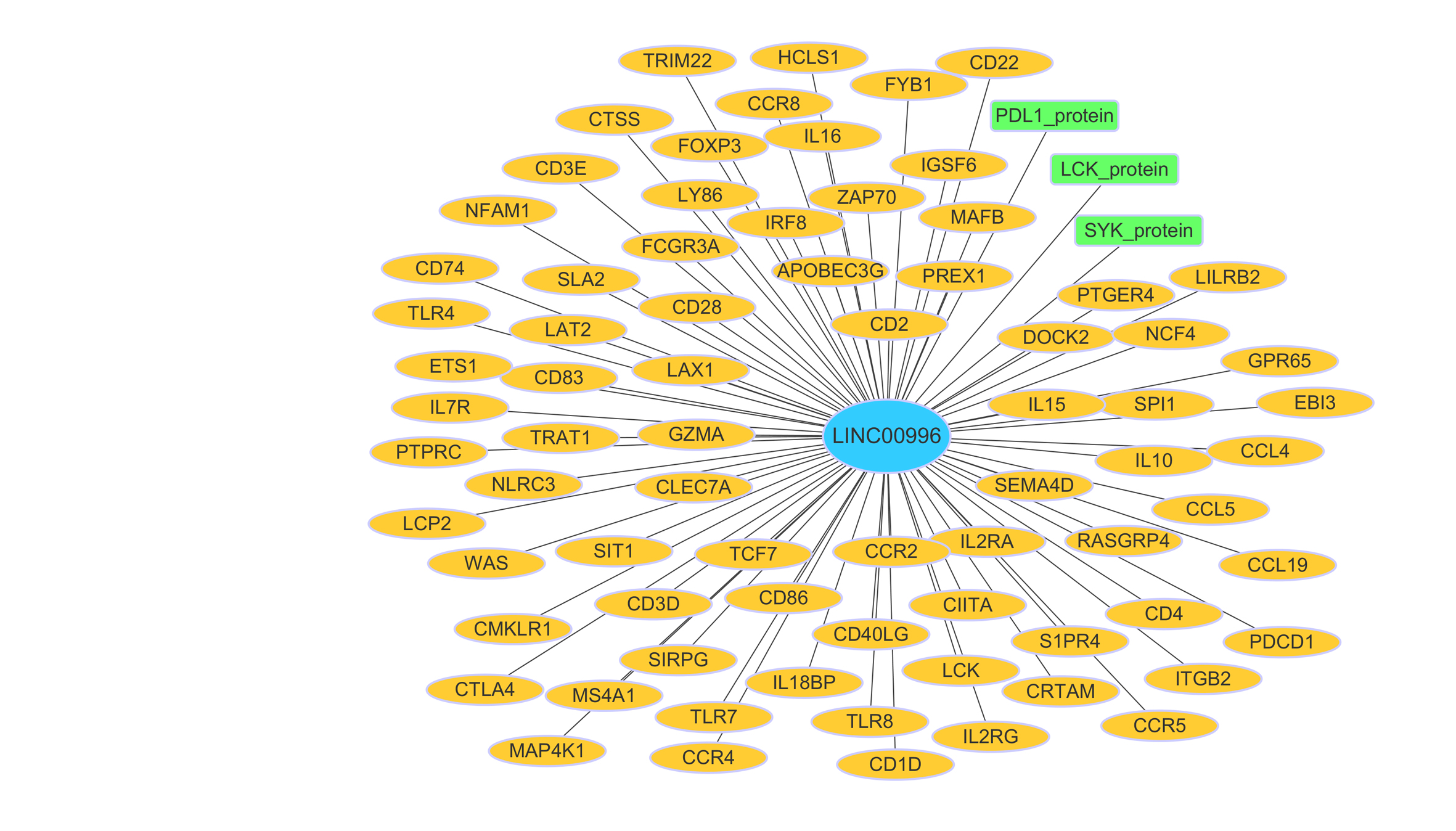

Supplement: Supplementary Figure 5 — Potential immune-related targets from TCPA (green round rectangle) and TCGA (orange ellipse) database of Linc0996 (blue ellipse). [file Image_5.jpeg]

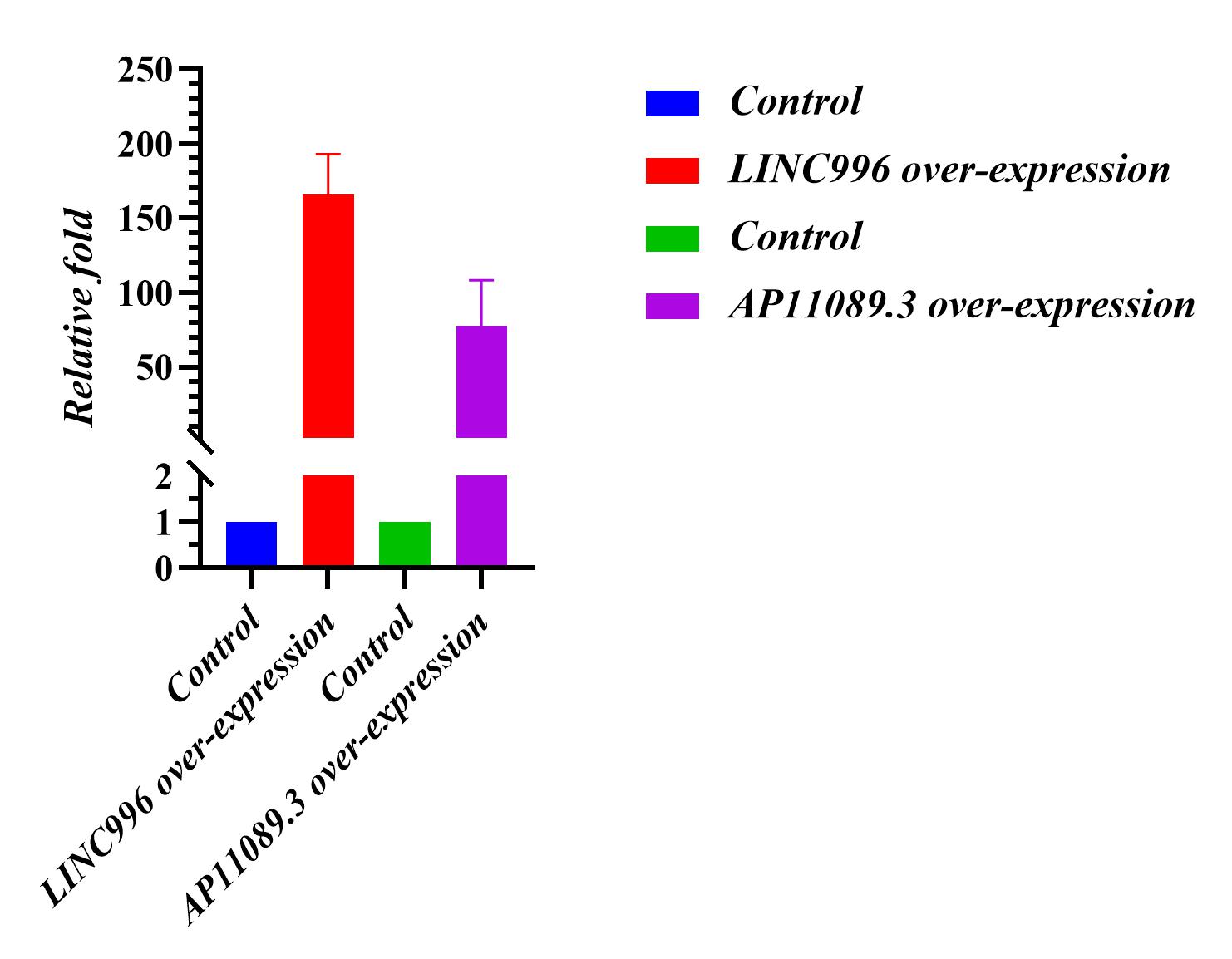

Supplement: Supplementary Figure 6 — PCR validated the achievement of LINC00996 and AP001189.3 transfection. [file Image_6.jpeg]

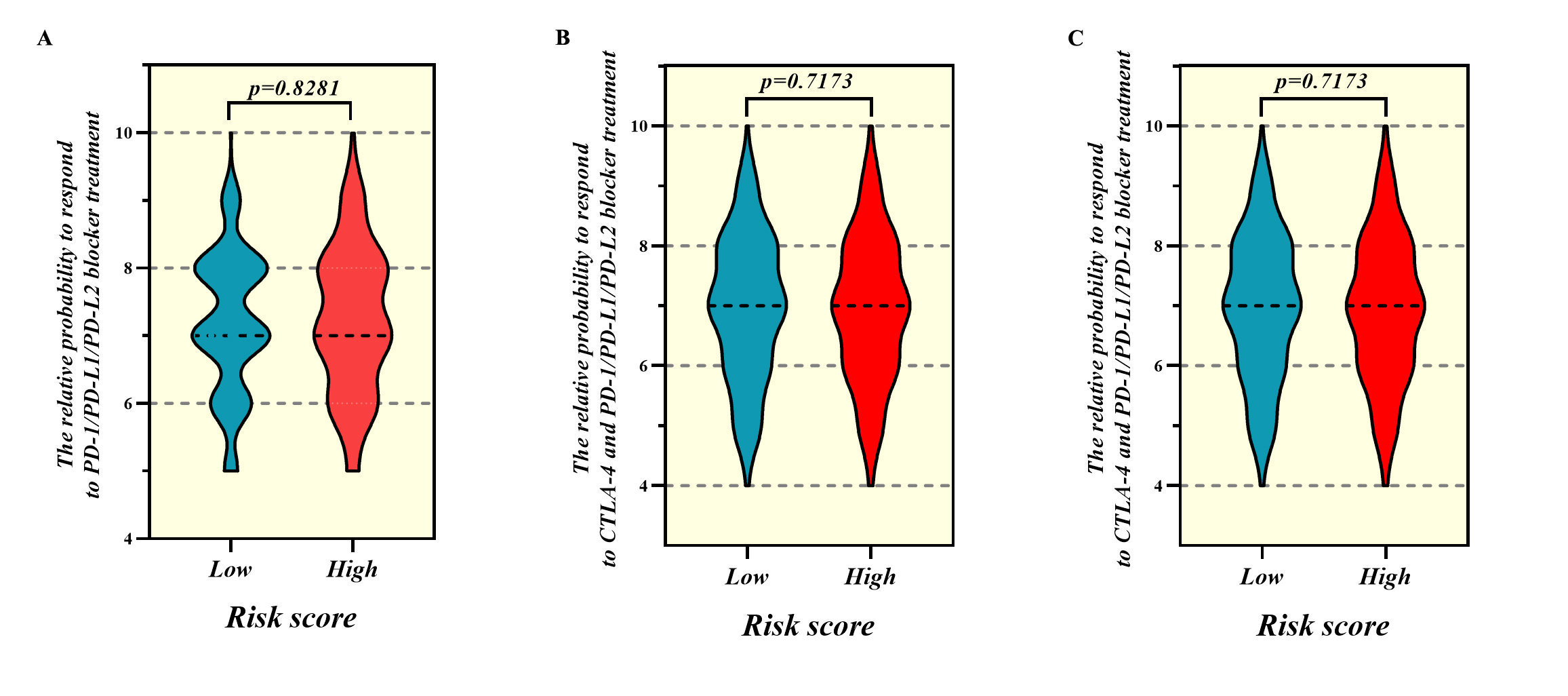

Supplement: Supplementary Figure 7 — The insignificant prediction result of ICI treatment between AD patients in high- and low-risk group. [file Image_7.jpeg]
