# Supplementary material for: The Immune Heterogeneity Between Pulmonary Adenocarcinoma and Squamous Cell Carcinoma: A Comprehensive Analysis Based on lncRNA Model
Source: Front Immunol. 2021 Jul 29;12:547333. doi: 10.3389/fimmu.2021.547333 (PMC8358782; doi:10.3389/fimmu.2021.547333)
Supplement: Supplementary file 8 [file DataSheet_1.zip › Data Sheet 1/Supp Mat/Supplementary Table 1.docx]

| **Table S1 Baseline characteristics of patients** | | | | |
| --- | --- | --- | --- | --- |
| Characteristic | AD | | SCC | |
|  | (n=434) | | (n=390) | |
| Age |  |  |  |  |
| Young (AD<71y; SCC<72y) | 294 | (67.4%) | 260 | (66.7%) |
| Old (AD≥71y; SCC≥72y) | 140 | (32.6%) | 130 | (33.3%) |
| Gender |  |  |  |  |
| Male | 200 | (46.1%) | 290 | (74.4%) |
| Female | 234 | (53.9%) | 100 | (25.6%) |
| Stage |  |  |  |  |
| I | 236 | (54.4%) | 183 | (46.9%) |
| II | 105 | (24.2%) | 131 | (33.6%) |
| III | 73 | (16.8%) | 70 | (18.0%) |
| IV | 20 | (4.6%) | 6 | (1.5%) |
| T |  |  |  |  |
| T1（≤3cm） | 152 | (35.0%) | 85 | (21.8%) |
| T2（≤5cm，＞3cm） | 229 | (52.8%) | 233 | (59.7%) |
| T3（≤7cm，＞5cm） | 36 | (8.3%) | 54 | (13.9%) |
| T4（＞7cm） | 17 | (3.9%) | 18 | (4.6%) |
| N |  |  |  |  |
| N0 | 285 | (65.7%) | 243 | (62.3%) |
| N1 | 84 | (19.4%) | 109 | (28.0%) |
| N2 | 63 | (14.5%) | 33 | (8.4%) |
| N3 | 2 | (0.4%) | 5 | (1.3%) |
| M |  |  |  |  |
| M0 | 296 | (68.2%) | 384 | (98.5%) |
| M1 | 20 | (4.6%) | 6 | (1.5%) |
| MX | 118 | (27.2%) | - | - |
| Status |  |  |  |  |
| Live | 286 | (65.9%) | 236 | (60.5%） |
| Death | 148 | (34.1%) | 154 | (39.5%） |
| Survival month | 793.69±40.557(33-6812) | | 949.9±45.655(30-5287) | |
