# Supplementary material for: The Immune Heterogeneity Between Pulmonary Adenocarcinoma and Squamous Cell Carcinoma: A Comprehensive Analysis Based on lncRNA Model
Source: Front Immunol. 2021 Jul 29;12:547333. doi: 10.3389/fimmu.2021.547333 (PMC8358782; doi:10.3389/fimmu.2021.547333)
Supplement: Supplementary file 8 [file DataSheet_1.zip › Data Sheet 1/Supp Mat/Supplementary Table 2.docx]

| Table S2 The Primer sequence in research | |
| --- | --- |
| Primer | Sequence（5'→3'） |
| AP001189.3-F | GAGTCTAGGTTCTGCTGCTTCC |
| AP001189.3-R | GGGTGTGGGCGATGATTATGA |
| AL589843.1-F | ATAAGCGGGCAGAGGGTAGAA |
| AL589843.1-R | CAGCATCGGTGAAGAGTGGAA |
| AL391121.1-F | GTCCCGATTTCTCTCCACCTCTG |
| AL391121.1-R | CGCCTTTCTGCCGACTTCTTC |
| SREBF2-AS1-F | TCTTCTGCTGCTTCTGCTTCTT |
| SREBF2-AS1-R | GCTTTCATGTCCAGGATCTCACT |
| AC245595.1-F | ACTCTACACCCTCTCTCCTTCTG |
| AC245595.1-R | CTCAGGTATCCAGGCACATTCAT |
| LINC00996-F | CTCTGCCACATCGTTCGGTTC |
| LINC00996-R | CTTCTTACGCTGCCAACTGCTAA |
| VIM-AS1-F | TCAGACCTGTAGCATCAGCATC |
| VIM-AS1-R | GGTTCTCAGAATCACCTGGTAAGT |
| SFTA1P-F | AGAAGGAGTGACTGAACATTGACA |
| SFTA1P-R | CTGGCTCTTGGTGAGATGGAT |
| MSC-AS1-F | CCCAACACCAGAGAACACTAAATG |
| MSC-AS1-R | CAATGCTGACATAGTCCTGAACAA |
| TMPO-AS1-F | AGGAGACGCCGATAAGGGACA |
| TMPO-AS1-R | CCAGAGACGAAAGCTGCTTCT |
| ABALON-F | ACAATCACCCAACACAACAGAAAG |
| ABALON-R | TGAATGACCACCTAGAGCCTTG |
| AC025048.4-F | GCTACCTTCTGCCTGGACTCA |
| AC025048.4-R | CCTTCCTCCTCCTGTGATCCT |
| LINC01138-F | ACATCAGCAGGAAGCAGTTACA |
| LINC01138-R | TCCTTCTGGTCTCCTACCTCATT |
| IPO5P1-F | CCAGCAACCAGGTGATGTGTT |
| IPO5P1-R | GGACCAGGCAGAAGAAGACAGT |
| AC008763.1-F | CAGCAGCCGTCTTGGATAACTT |
| AC008763.1-R | GGAACATTGGGCAGGACTGAAT |
| AL606489.1-F | ACTCAGGAAAGACATCAGCAGAG |
| AL606489.1-R | AGGCATTGTGAAATAGGCAAGAAG |
| AC026355.1-F | CGTGGTAATCTGAGTGTGGTAAGC |
| AC026355.1-R | GACCGATATGCCTATGCGAATGT |
| AC123595.1-F | TCGCAGGACTTCGCAGATACC |
| AC123595.1-R | CGCTCGGTCGGGATTCAAAAG |
| 18SrRNA-F | AAACGGCTACCACATCCAAG |
| 18SrRNA-R | CCTCCAATGGATCCTCGTTA |
