# Supplementary material for: The Immune Heterogeneity Between Pulmonary Adenocarcinoma and Squamous Cell Carcinoma: A Comprehensive Analysis Based on lncRNA Model
Source: Front Immunol. 2021 Jul 29;12:547333. doi: 10.3389/fimmu.2021.547333 (PMC8358782; doi:10.3389/fimmu.2021.547333)
Supplement: Supplementary file 8 [file DataSheet_1.zip › Data Sheet 1/Supp Mat/Supplementary Table 3.docx]

| **Table S3 Cross table for low-risk and high-risk group of AD patients** | | | | | | | | |
| --- | --- | --- | --- | --- | --- | --- | --- | --- |
| Characteristic | Low Risk | | | | High Risk | | | p Value |
|  | (n=146) | (n=161) | | | | | |  |
| Age |  | |  |  | |  |  | |
| Young(<71y) | 111 | 101 | | | | | 0.013 | |
| Old(≥71y) | 35 | 60 | | | | |  |  |
| Gender |  |  | | | | |  | |
| Male | 69 | 79 | | | | | 0.819 | |
| Female | 77 | 82 | | | | |  |  |
| Stage |  |  | | | | |  | |
| I | 90 | 69 | | | | | 0.003 | |
| II | 32 | 41 | | | | |  |  |
| III | 16 | 39 | | | | |  |  |
| IV | 8 | 12 | | | | |  |  |
| T |  |  | | | | |  | |
| T1（≤3cm） | 54 | 39 | | | | | 0.023 | |
| T2（≤5cm，＞3cm） | 78 | 95 | | | | |  |  |
| T3（≤7cm，＞5cm） | 6 | 18 | | | | |  |  |
| T4（＞7cm） | 8 | 9 | | | | |  |  |
| N |  |  | | | | |  | |
| N0 | 105 | 90 | | | | | 0.023 | |
| N1 | 27 | 37 | | | | |  |  |
| N2 | 13 | 34 | | | | |  |  |
| N3 | 1 | - | | | | |  |  |
| M |  |  | | | | |  | |
| M0 | 138 | 149 | | | | | 0.321 | |
| M1 | 8 | 12 | | | | |  |  |
