# Supplementary material for: The Immune Heterogeneity Between Pulmonary Adenocarcinoma and Squamous Cell Carcinoma: A Comprehensive Analysis Based on lncRNA Model
Source: Front Immunol. 2021 Jul 29;12:547333. doi: 10.3389/fimmu.2021.547333 (PMC8358782; doi:10.3389/fimmu.2021.547333)
Supplement: Supplementary file 8 [file DataSheet_1.zip › Data Sheet 1/Supp Mat/Supplementary Table 4.docx]

| **Table S4 Cross table for low-risk and high-risk group of SCC patients** | | | | | | |
| --- | --- | --- | --- | --- | --- | --- |
| Characteristic | Low Risk | | High Risk | | | p Value |
|  | (n=196) | | (n=191) | | |  |
| Age |  |  | |  |  |  |
| Young(<72y) | 139 | | 121 | | | 0.13 |
| Old(≥72y) | 57 | | 70 | | |  |
| Gender |  | |  | | |  |
| Male | 152 | | 136 | | | 0.094 |
| Female | 44 | | 55 | | |  |
| Stage |  | |  | | |  |
| I | 94 | | 88 | | | 0.879 |
| II | 62 | | 68 | | |  |
| III | 37 | | 32 | | |  |
| IV | 3 | | 3 | | |  |
| T |  | |  | | |  |
| T1（≤3cm） | 42 | | 43 | | | 0.381 |
| T2（≤5cm，＞3cm） | 124 | | 107 | | |  |
| T3（≤7cm，＞5cm） | 22 | | 32 | | |  |
| T4（＞7cm） | 8 | | 9 | | |  |
| N |  | |  | | |  |
| N0 | 117 | | 125 | | | 0.718 |
| N1 | 58 | | 49 | | |  |
| N2 | 18 | | 15 | | |  |
| N3 | 3 | | 2 | | |  |
| M |  | |  | | |  |
| M0 | 193 | | 188 | | | 0.645 |
| M1 | 3 | | 3 | | |  |
